# Hidden cost of hospital-based delivery and associated factors among postpartum women attending public hospitals in Gamo zone, southern Ethiopia

**DOI:** 10.1186/s12913-024-10927-y

**Published:** 2024-04-22

**Authors:** Menen Tilahun Chewaka, Gistane Ayele, Godana Yaya Tessema, Dagne Deresa Dinagde, Hana Tadesse Afework, Bezalem Mekonen Biwota, Habtamu Wana Wada

**Affiliations:** 1 Department of Midwifery, College of Health Sciences, Mizan Tepi University, Mizan Tepi, Ethiopia; 2 Department of Midwifery, College of Medicine and Health Sciences, Arba Minch University, Arba Minch, Ethiopia; 3 Departments of Midwifery, College of Health Sciences, Mattu University, Mettu, Ethiopia; 4 Department of Midwifery, Arba Minch Health Sciences College, Arba Minch, Ethiopia

**Keywords:** Hidden costs, Hospital costs, Hospital -based delivery, Ethiopia

## Abstract

**Background:**

Since 2005, the healthcare system in Ethiopia has implemented policies to promote the provision of free maternal healthcare services. The primary goal of these policies is to enhance the accessibility of maternity care for women from various socioeconomic backgrounds. Additionally, the aim is to increase the utilization of maternity services, such as institutional deliveries, by removing financial obstacles that pregnant women may face. Even though maternity services are free of charge. The hidden cost has unquestionably been a key obstacle in seeking and utilizing health care services. Significant payments due to delivery services could create a heavy economic burden on households.

**Objectives:**

To determine the hidden cost of hospital-based delivery and associated factors among postpartum women attending public hospitals in Gamo zone, southern Ethiopia 2023.

**Methods:**

A facility-based cross-sectional study was conducted on 411 postpartum women in Gamo Zone Public Health Hospitals from December 1, 2022, to January 30, 2023. The systematic sampling technique was applied to reach study units. Data was collected using the Kobo Toolbox Data Collection Tool and exported to SPSS statistical software version 27 for analysis. Simple linear regression and multiple linear regression were done to see the association of variables. The significance level was declared at a P-value < 0.05 in the final model.

**Result:**

The median hidden cost of hospital-based delivery was 1142 Ethiopian birr (ETB), with a range (Q) of 2262 (504–2766) ETB. Monthly income of the family (β = 0.019), obstetrics complications (β = 0.033), distance from the health facility (β = 0.003), and mode of delivery (β = 0.072), were positively associated with the hidden cost of hospital-based delivery. While, rural residence (β = −0.041) was negatively associated with the outcome variable.

**Conclusion:**

This study showed the hidden cost of hospital based delivery was relatively high. Residence, monthly income of the family, obstetric complications, mode of delivery, and distance from the health facility were statistically significant. It is important to take these factors into account when designing health intervention programs and hospitals should prioritize the availability of essential drugs and medical supplies within their facilities to address direct medical costs in hospitals.

**Supplementary Information:**

The online version contains supplementary material available at 10.1186/s12913-024-10927-y.

## Introduction

The hidden costs of hospital-based delivery include costs that include the purchase of outside medications, food and drink, lodging, transportation, communication, and loss of wages by both the patient and the patient’s companions during the hospital stay, which were estimated from the total direct medical cost, direct non-medical cost, and total indirect cost. Direct medical costs include spending for diagnosis, medical supplies, drugs, and consultation. Direct non-medical costs indicate expenditures for food, accommodation, and transportation for the patient as well as for accompanying individuals. Indirect costs are caused by absence from work and, thereby, productivity loss [1, 2].

Free maternity healthcare services have been implemented in various parts of the world to enhance access to maternity care for women from diverse socioeconomic backgrounds [3]. As well as to increase the number of women seeking maternity services like antenatal care and institutional delivery by eliminating the financial barriers among pregnant women [3, 4]. The government of Ethiopia recommends that every woman give birth with the assistance of skilled birth attendants. To achieve this, FMOH has recognized that maternity services have been made free of charge in the country [5, 6].

Improving maternal health is also the main concern of the WHO (World Health Organization), as every day approximately 810 women die from childbirth and related problems that are preventable [7]. This death occurs most commonly in middle- and low-income countries. In 2017, about 295,000 women died because of pregnancy and childbirth-related complications. Of these, 94% of deaths occurred in low and middle-income countries, and Sub-Saharan Africa alone accounted for about two-thirds (196,000) of maternal deaths [8].

In Ethiopia, according to the EDHS 2016 report, the maternal mortality rate was 412 per 100,000 live births, which is the highest rate in sub-Saharan Africa. The majority of complications leading to maternal mortality in Ethiopia are attributed to factors such as limited utilization of skilled care and institutional delivery, home births, restricted access to health services, inadequate quality of care, long distances to healthcare facilities, lack of information, and poverty. Economic constraints faced by women seeking care at public health facilities have a significant impact on the utilization of health services [2, 9–11].

Therefore, hidden costs have unquestionably been a key obstacle to seeking and utilizing health care services. Significant payments due to delivery services could create a heavy economic burden on households. For instance, a study conducted here in Ethiopia shows that the hidden cost was 877.5 ETB, which is difficult to afford for poor households. This shows that hidden costs play an important role in the low utilization of facility-based deliver [1, 12–15].

The government of Ethiopia announced free maternity health care service utilization in government facilities. This policy aims to ensure financial protection and improve the utilization of health services, especially for low-income people [16]. Hospital expenses, including medications (if in stock), registration, consultations, and beds, are all legally covered under the free service program. Yet, because of a scarcity of certain supplies and medications, patients are frequently directed to independent pharmacies and required to pay out-of-pocket to medical costs [17, 18].

Based on the available information, no prior studies have been conducted in the specific study area. Even in Ethiopia, there is only one study that has explored the extent of hidden costs among postpartum women attending hospitals and the factors associated with these costs. Consequently, this study holds significant importance as it contributes to expanding the existing literature by examining the impact of hidden costs resulting from hospital-based childbirth on household economies in diverse sociocultural contexts. This study was conducted to assess the hidden costs during hospital delivery and to determine the factors associated with the hidden costs of hospital-based delivery in the Gamo Zone Public Hospital by filling the time and place gaps.

## Method and materials

### Study area and setting

The study was conducted at Gamo zone public hospitals among the Ethiopian southern zones Arba-Minch Town serves as the administrative center of Gamo Zone. It is situated approximately 495 km to the south of Addis Ababa, the capital city of Ethiopia, and around 275 km southwest of Hawassa, commercial center of southern peoples region. Based on the 2007 Census conducted by the Central Statistical Agency of Ethiopia (CSA), this zone has a total population of 1,659,310, of whom 779,332 are men and 879,782 women [19]. In terms of health care coverage, Gamo zone has 63 public health facilities in which 57 are health center sand 6 hospitals namely Arba Minch general hospital, Dilfana primary hospital, Kamba primary hospital, Gerese primary hospital, Chencha primary hospital, and Selam Ber primary hospital. All these hospitals provide inpatient and outpatient medical services, surgical procedures, antenatal care, intrapartum care, and postnatal care, immunization, pediatric, and family planning services.

### Study design and period

A facility based cross sectional study was conducted among post-partum women attending post natal care at Gamo zone public hospitals, southern Ethiopia from December 1, 2022 -January 30, 2023.

### Study participants

The source population of this study consisted of women who had given birth in the selected hospitals and those who were present and willing to participate during the data collection period. The study population was then selected through systematic sampling from this source population. The data was collected from individuals actively involved in the process of purchasing, such as caregivers, family members, or partners, who served as the study units. Mothers who were severely ill and not ready for discharge on the day of data collection, as well as participants who were referred to another hospital for further investigation and management, were excluded from the study.

### Sample size determination

Single populations mean formula was used to determine the number of study participants. The standard deviation were derived from a study conducted in Bale Zone, Southeast Ethiopia to estimate the sample size [1]. Accordingly, the standard deviation 5, margin of error 0.49, at- 95% confidence interval, and 5% non-response rate were considered to estimate the sample size. Hence, the required sample size was calculated as;

$${\rm{n}}\,{\rm{ = }}\,\,{{{{\left( {{\rm{z}}{{\rm{\alpha }} \mathord{\left/{\vphantom {{\rm{\alpha }} {\rm{2}}}} \right.\kern-\nulldelimiterspace} {\rm{2}}}} \right)}^{\rm{2}}}{{\rm{\delta }}^{\rm{2}}}} \over {{{\rm{d}}^{\rm{2}}}}}$$

Where n = required sample size,

${\left( {{{\rm{Z}}_{{{\rm{\alpha }} \over {\rm{2}}}}}} \right)^{\rm{2}}}$ = reliability coefficient for 95% confidence interval  (1.96),

δ = standard deviation (5).

d = assumed margin of error (0.49), d = Z* (σ / √n), from previous study.

$${\rm{n = }\,}{{{{\left( {{\rm{1}}{\rm{.96}}} \right)}^{\rm{2}}}{{\left( {\rm{5}} \right)}^{\rm{2}}}} \over {{{\left( {{\rm{0}}{\rm{.49}}} \right)}^{\rm{2}}}}}$$

Based on the assumptions by adding 10% non-response rate the final calculated sample was: *N* = 420.

### Sampling procedure and technique

**Fig. 1:**
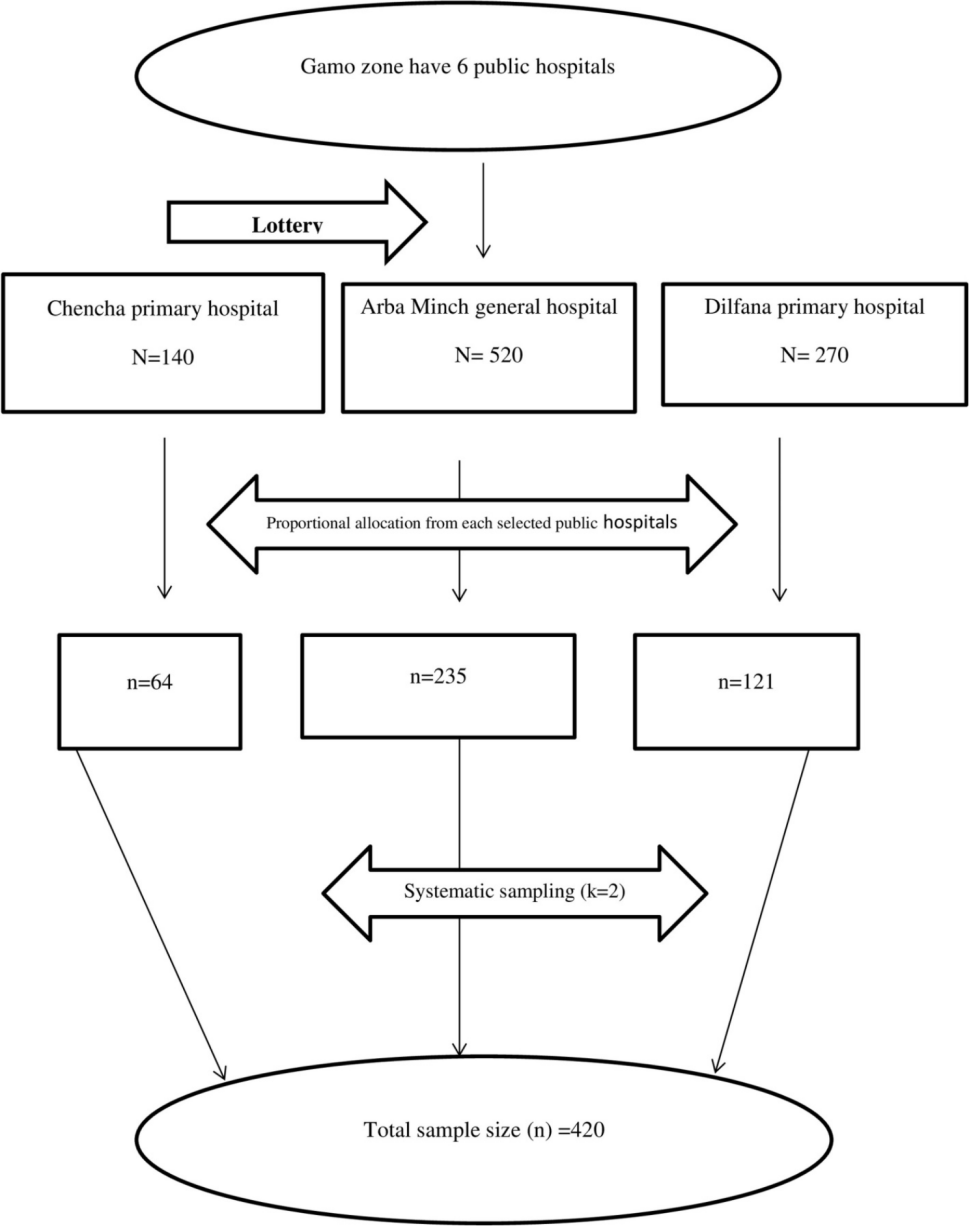
Schematic presentation of proportionate fraction of sample size of each hospital of Gamo zone among postpartum women attending public hospitals, 2023

There were six hospitals in Gamo Zone, namely: Arba Minch general hospital, Gerese, Kamba, Chencha, Dilfana, and Selam-Ber primary hospitals. Among these six hospitals, three hospitals, namely Arba Minch general hospital, Chencha primary hospital, and Dilfana and primary hospital, were selected randomly by lottery. Study participants were selected by a systematic sampling technique, using the last year’s annual delivery report of the similar two months of the study period as the sample frame. Thus, delivery reports for the same months last year at Arba Minch general hospital, Chencha, and Dilfana primary hospitals were 520, 270, and 140, respectively. Then the proportional allocation of samples was done for each hospital. Accordingly, 235 samples came from Arba Minch general hospital, 121 samples came from Dilfana primary hospital, and 64 samples came from Chencha primary hospital. To have an individual study participant from each hospital, a systematic sampling technique was used during the data collection process with a K value of 2 (that means at intervals of two women after delivery during the discharge) (Fig. 1).

Proportional allocation formula: ${\rm{nj = }\,}{{{\rm{Nj}}} \over {\rm{N}}}{\rm{n}}$,

Where, nj = number of units or individuals within a specific subgroup or stratum,

N = the size of the entire population,

Nj = Size of Subgroup and.

n = the desired sample size for the study or survey [20, 21].

### Study variable

#### Dependent variable

- Hidden cost of hospital based delivery.

#### Independent variables

- Socio-demographic factors: includes age, residence, educational status of mother and husband, occupation of mother and husband and family monthly income.
- Access to health service: includes length of stay in hospital, distance of hospital availability of transportation, place of delivery.
- Delivery related: factors include number of delivery, obstetrics complications and mode of delivery.

### Operational definition and definition of terms

#### The hidden cost of hospital-based delivery

The costs of hospital-based delivery service include the purchase of outside medications, food and drink, lodging, transportation, communication, and loss of wages by both the patient and the patient’s companions during the hospital stay, which was estimated from total direct medical cost, direct non-medical cost, and total indirect cost [1]. The data regarding the expenses incurred for various purchases was collected by requesting patients to present receipts and by inquiring attendants or caregivers about the amounts they spent on different purchases.

#### Direct medical cost

An amount of any money that has to be paid by mothers for direct medical costs that are out of stock in the study hospitals during the study period [1, 2].

#### Direct non-medical costs

An amount of any money that has to be paid for transportation, food, communication, and accommodation costs for both patients and caretakers to visit the study facility during the study period [1, 22].

#### Indirect cost

wage loss of self and/or caretaker and cost that incurred due to an institutional stay and which is calculated using the human capital approach [2].

$$\eqalign{& {\rm{Income}}{\mkern 1mu} \,{\rm{loss}}{\mkern 1mu} \,{\rm{of}}\,{\mkern 1mu} {\rm{employed}} \cr & = {{{\rm{previous}}\,{\mkern 1mu} {\rm{monthly}}{\mkern 1mu} \,{\rm{income}}\left( {{\rm{ETB}}} \right)} \over {\# {\mkern 1mu} {\rm{of}}{\mkern 1mu} \,{\rm{working}}\,{\mkern 1mu} {\rm{day}}{\mkern 1mu} \,{\rm{in}}{\mkern 1mu} \,{\rm{the}}{\mkern 1mu} \,{\rm{month}}}}*{\rm{length}}\,{\mkern 1mu} {\rm{of}}\,{\mkern 1mu} {\rm{stay}} \cr} $$

$$\eqalign{& {\rm{Income}}{\mkern 1mu} \,{\rm{loss}}{\mkern 1mu} \,{\rm{of}}{\mkern 1mu} {\rm{\,unemployed}} \cr & = {{{\rm{self - reported}}{\mkern 1mu} \,{\rm{daily}}/{\rm{replacement}}\,{\mkern 1mu} {\rm{income}}} \over {\# {\mkern 1mu} {\rm{\,of}}{\mkern 1mu} {\rm{\,working}}\,{\mkern 1mu} {\rm{day}}{\mkern 1mu} \,{\rm{in}}{\mkern 1mu} \,{\rm{the}}{\mkern 1mu} \,{\rm{month}}}}\,*{\rm{length}}{\mkern 1mu} \,{\rm{of}}{\mkern 1mu} \,{\rm{stay}} \cr} $$

**Obstetrics complications** are an acute condition arising from a direct cause of maternal death, such as postpartum hemorrhage, obstructed labor, postpartum sepsis, and postpartum eclampsia and preeclampsia [23].

### Data quality control

To assure the quality of the data, the questionnaires were translated from English to Amharic and retranslated to English for a consistent and proper check. Two weeks prior to the actual data collection period, a pre-test was done on 5% (21) of the computed sample at wolaita Sodo university comprehensive and specialized hospital, which has similar socio-cultural and living standard with study area. Necessary feedback and adjustment on the phrasing were done accordingly. Two-day training was given to data collectors to become familiar with the data collection tool. The completed questionnaires were cross-checked on a daily basis. To ensure the completeness of information during data collection, the principal investigator and supervisor made a thorough check.

### Data analysis and entry

The data was collected by Kobo Toolbox software and imported to the Statistical Package for Social Science (IBM SPSS) Window Version 27 for coding, cleaning, and analysis. Missing values were checked by running frequencies and other data explorations. The responses in the completed questionnaire were exported to IBM SPSS version 27 for analysis. Multiple linear regressions were used to see the association between each independent variable and the dependent variable. Descriptive analysis was used to clean the data. Errors related to the inconsistency of data were checked and corrected during data cleaning. Prior to conducting the multiple linear regression analysis, all assumptions were examined. The normality of the data distribution was assessed using a Q-Q plot, which revealed that the points on the plot did not exhibit a close alignment along a straight line. This departure from a straight line indicates a deviation from the assumption of normality in the data distribution. To assess the relationship between the independent variables, a check for multicollinearity was conducted. Variables with a Variance Inflation Factor (VIF) of less than 10 were deemed acceptable, indicating no significant collinearity among the variables. Based on this information, it was concluded that the data were reasonably regularly distributed, hence no changes were necessary. Dummy variables were created for the categorical variables. The main outcome variable, the total hidden cost of facility-based delivery, was estimated by adding the direct nonmedical cost and the indirect and direct medical costs. Descriptive statistics, including percentage and frequency, were used to portray the socio-demographic and delivery-related information of the study participants. The median and mean were estimated to show the hidden cost of hospital-based delivery. To examine factors associated with the outcome variable, first, bivariate linear regression analyses were conducted. Then, those covariates with p-values of less than 0.25 were included in the multivariate regression model. Adjusted R_2_ was used for the ability of explanatory variables to explain dependent variables. The hidden costs of hospital-based delivery were predicted using unstandardized ß-coefficients. Finally, statistical significance was determined at p-values less than 0.05 and with a 95% CI.

## Results

### Socio demographic characteristics of respondents

A total of 411 postpartum women who delivered at the selected hospitals participated in the study, yielding a 97.8% response rate. Five participants declined to give consent as they rushed to leave, and four of them discontinued the interview because they were destroyed by their baby’s. About 161 (39.2%) of the participants were in the age group of 20–24 years. The mean age of the respondents was 26.13 (SD ± 4.1) years. The majority of the respondents were Orthodox Christians, 196 (47.7%). About 234 (56.9%) of the women were from rural areas, and 142 (34.5%) of the respondents were passed through a referral system. The median family incomes of respondents were 3200 ETB with an SD of 2403. In terms of educational attainment, a majority of 176 (42.8%) of our respondents attended no formal education. A majority of the 209 (50.9%) study participants were housewives. 112 (27.3%) of respondents’ husbands attended more than secondary school, and 150 (36.5%) of respondents’ husbands were farmers (Table 1).

**Table 1:** Socio-demographic characteristics of the study participants in southern Ethiopia, 2023 (*N* = 411)

Variables	Categories	Frequency	Percent
Age of respondent	20–24 25–29 30–34 >=35	161 160 60 30	39.2 38.9 14.6 7.3
Residence of respondents	Urban Rural	177 234	43.1 56.9
Method of arrival	Referral By self	142 269	34.5 65.5
Level of education mother	No formal education Primary education Secondary education More than secondary education	176 96 81 58	42.8 23.4 19.7 14.1
Occupation of mother	House wife Government employee Merchant private employee	209 82 66 54	50.9 20.0 16.1 13.0
level of education of father	No formal education Primary education secondary education more than secondary education	110 90 99 112	26.7 21.9 24.1 27.3
Occupation of father	Farmer Government employee Merchant private employee	150 115 114 32	36.5 28.0 27.7 7.8
Monthly income of family	< 1000 1001–3000 3001–5000 5001–7000 > 7000	103 102 104 71 31	25.1 24.8 25.3 17.3 7.5
Religion	Orthodox Protestant Muslim Others *	196 133 58 24	47.7 32.4 14.1 5.8

*Key notes* *Indicates catholic, Adventist

### Delivery–related and access to health service information

Of all the study participants, 287 (69.8%) mothers had vaginal deliveries, and 362 (88%) were multigravida mothers. Among the study participants, 232 (56.4%) gave birth at general hospitals, and 127 (30.9%) faced different obstetric complications. About 153 (37.2%) respondents used an ambulance, while 157 (38.0%) study participants used a Bajaj to come to the hospital. The mean length of stay in the hospital was 2.3 days and ranged from 1 to 6 days, and the median distance from the healthy facility was 10 km (Table 2).

**Table 2:** Delivery related and access to health service information of the study participants in southern Ethiopia, 2023 (*N* = 411)

Variable	Categories	Frequency	Percent
Mode of delivery	Cesarean section Vaginal delivery	124 287	30.2 69.8
Para	Primipara Multi- Para Grand multi-Para	49 308 54	11.9 74.9 13.2
Place of delivery	General hospital Primary hospital	232 179	56.4 43.6
Obstetrics complication	Yes No	127 284	30.9 69.1
Method of transportation	Ambulance private car Bajaj others *	153 42 157 59	37.2 10.2 38.2 14.4

* = locally made stretcher, on foot, public transportation

### Estimation of hidden cost of hospital deliveries

The median hidden cost of hospital-based delivery was 1142 Ethiopian birr (ETB), with a range (Q) of 2262 (504–2766) ETB. The median direct medical cost of hospital-based delivery was 455 ETB, while the direct nonmedical costs incurred by women were 330 ETB. And the median loss of wages that mother and caretaker incurred due to the hospital stay was about 350 ETB), see (Table 3).

**Table 3:** Hidden cost of hospital based delivery of the study participants in southern Ethiopia, 2023 (*N* = 411)

Type of cost	Median ETB	Min–max cost(ETB)	standard deviation (± SD)
**Direct medical cost**	455.00	100–1120	196
Cost of drug and other medical supply	455.00	100–1120	196
**Direct non-medical cost**	330.00	110–905	140
Food expenses	150.00	100–270	54
Drinking expenses	120.00	30–300	56
Transport expenses	150.00	50–700	105
communication expense	10.00	10–100	23
**Indirect cost**	350.00	98-1833.3	307

### Hidden cost among included hospitals

**Fig. 2:**
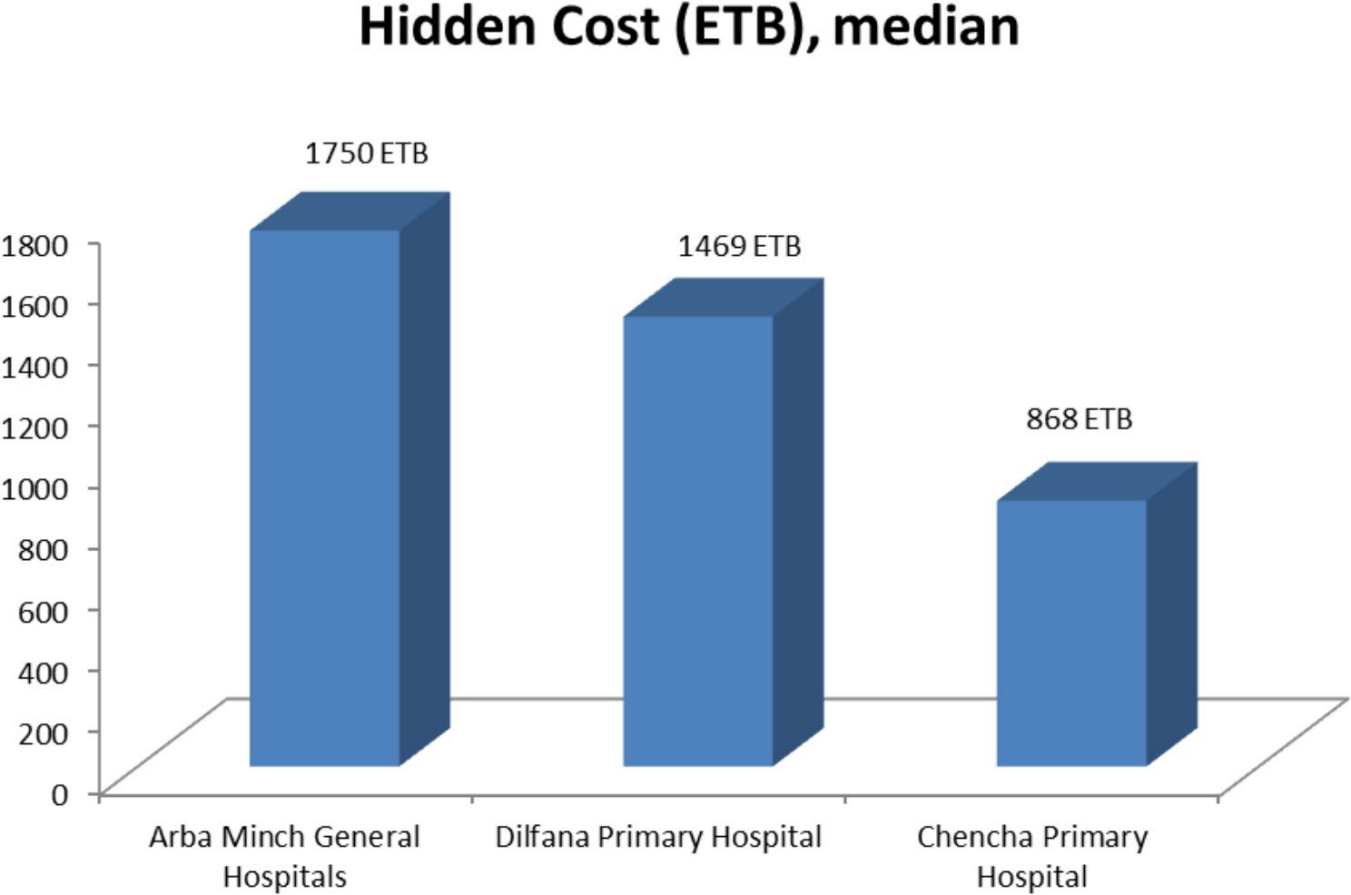
Hidden cost of institutional based delivery among included hospitals, southern Ethiopia, 2023

The respondents from Arba Minch General Hospital reported significantly higher hidden costs associated with hospital-based delivery compared to the two primary hospitals, namely Chencha Hospital and Dilfana Hospital (Fig. 2).

### Multiple linear regression analysis result

Multiple linear regressions were employed to see the association between the possible explanatory variables and the outcome variable (the hidden cost of hospital-based delivery). Age, residence, distance from the healthy facility, educational status of the father,, monthly income of the family, length of hospital stay, mode of delivery, method of arrival, and obstetric complications were significantly associated with outcomes of the hidden cost of hospital based delivery in simple linear regression at a p-value less than 0.25. However, in the multiple linear regression analysis, residence, monthly income of the family, obstetric complications, distance from the healthy facility, and mode of delivery) were found to be statistically significant at a p value < 0.05. The constructs of the model explained that the independent variable explained the dependent variable by 31.1% (Adjusted R2 = 0.311, *P* < 0.001). On another hand, the interpretation of the multiple linear regression model with an adjusted R-squared of 0.311 and a p-value less than 0.001 indicates that the independent variables included in the model explain approximately 31.1% of the variation in the dependent variable, and the overall relationship between the independent variables and the dependent variable is statistically significant.

In this model, as distance increased by 1 km, the hidden cost of hospital based delivery increased by 0.003 ETB (β = 0.003, 95% CI = 0.001, 0.004), holding other variables constant. Women with obstetric complications had a higher hidden cost of hospital-based delivery compared to those with no obstetric complications by 0.033 ETB (ß =0.033; 95% CI = 0.005–0.062).

In this study, the monthly income of the family was found to have a statistically significant association with the hidden cost of hospital-based delivery. The results indicate that for every 1 birr increase in monthly income, the hidden cost of hospital-based delivery increased by 0.019 ETB (ß = 0.019; 95% CI = 0.007, 0.031). Women who delivered by cesarean section increased the hidden cost of hospital-based delivery by 0.072 ETB compared to those who delivered vaginally (ß = 0.072, 95% CI = 0.035, 0.109).

Rural residence has a negative association with the hidden cost of hospital-based delivery. The hidden cost of mothers from rural residence was reduced by 0.041ETB (ß=-0.041, 95%CI=-0.072, -0.011) as compared to mothers from urban residence (Table 4).

**Table 4:** Multiple linear regression analysis of hidden cost of hospital based delivery and associated, 2023 (*N* = 411)

Variable	Category	β	SE	*p*-value	95%CI	
Constant		2.934	0.049	0.001	2.837	3.031
Residence	rural urban (ref)	−0.041	0.016	0.012*	0.072	0.011
family monthly income		0.019	0.006	0.001*	0.007	0.031
obstetrics complication	Yes No(ref)	0.033	0.014	0.023*	0.005	0.062
distance from the health facility		0.003	0.001	0.001*	0.001	0.004
mode of delivery	cesarean section vaginal delivery (ref)	0.072	0.019	0.001*	0.035	0.109
length of stay		0.022	0.011	0.055	− 0.001	0.044
age of respondent		0.002	0.002	0.197	− 0.001	0.005
educational status of husband	no formal education	− 0.050	0.024	0.041	− 0.098	− 0.002
	primary education	0.016	0.023	0.490	− 0.029	
	secondary education	0.012	0.019	0.547	− 0.027	0.060
	above secondary education(ref)					
method of arrival	self referred from health facility(ref)	0.001	0.016	0.964	− 0.031	0.033

* = significance at *p*-value < 0.05

## Discussion

The median hidden cost of hospital based delivery was 1142 ETB. This was higher than study conducted in Bale Zone, Southeast Ethiopia, in which the hidden cost was 877.5 ETB [1]. The possible explanation for the discrepancy might be due to the difference in the cost of medication, food and drinking, transportation, informal payments, and difference in price due to current inflation. Again, this may be due to the Ethiopian government’s budget constraints resulting from civil war and economic crises have led to a scarcity of funds for the provision of medical materials, including drugs. As a result, patients are compelled to purchase these items from private pharmacies.

This study revealed that the direct medical cost of hospital-based delivery was 455 ETB. This result is higher than that of a study done in south-western Ethiopia, where it was suggested that the median cost was 310.8 ETB [1]. This might be due to the difference in the cost of drugs and medical supplies. Additionally, the studies might have been conducted at different time points, and the cost of healthcare services could have increased due to inflation or other economic factors.

In this study, direct non-medical costs make up a smaller percentage than direct medical costs. This outcome differed from a study conducted in Nepal, where the cost of dietary expenses was a significant factor in the price of hospital-based delivery [2]. The cause of this could be underreporting because most relatives in our country visit women in hospitals with homemade foods and drinks that are not considered costs. Furthermore, over time, inflation and economic changes can lead to increases in the cost of medical services and supplies. Since there was a time gap between the two studies, it is possible that the cost of hospital-based delivery has risen in the intervening period.

This study found that the total median indirect cost was 350 ETB. This finding was lower than the study done in Mekele General Hospital, Northern Ethiopia, in which the wage losses were 1030.95 ETB [24]. The possible reason might be an increase in the length of stay in the hospital. The average length of stay in this study is 2.3 days, resulting in a daily cost increase that inflates the daily wage loss.

This study revealed that the hidden cost of hospital-based delivery for mothers who delivered through a cesarean section was higher as compared to mothers who delivered vaginally. The finding was supported by a study conducted in Nepal and India [2, 25]. This is clear given that caesarean sections require more complicated procedures, specialized medical equipment, and longer hospital stays than other modes of delivery.

This study revealed that being away by 1 km from the hospital will result in an increment of 0.003 ETB of the hidden cost of hospital-based delivery. This is in line with a cross-sectional study conducted in Nepal, south-west Ethiopia, India, and Zimbabwe [1, 2, 26–28]. The possible explanation might be that it is obvious that distance and cost are directly correlated; as distance increases, so do prices. Also, living in an area with poor road infrastructure has a significant impact on cost.

It is found that those with higher incomes are more likely to spend on hidden costs. This is similar to cross-sectional studies conducted in Mekele, south-west Ethiopia, India and Nepal [1, 2, 24, 25]. This is because mothers with better incomes can afford to use services whenever needed. They may also prefer quality care and branded drugs, even if the prices are expensive.

This study also showed that there was a statistical association between residence and the hidden cost of hospital-based delivery. This is supported by a cross-sectional study conducted in north-west Ethiopia [29]. The possible reason might be that it is obvious that those who came from urban areas used to prefer high-standard care, which demands a high cost.

This study showed that women with obstetric complications had a high hidden cost of hospital-based delivery compared to those who had no obstetric complications. It is obvious that having obstetric complications requires extensive, life-saving curative and rehabilitative care, both of which are more expensive.

Living in a rural area is negatively correlated with the hidden cost of hospital-based delivery. Mothers residing in rural areas experienced a reduction of 0.041 Ethiopian Birr in hidden costs compared to mothers from urban areas. This difference may be attributed to financial limitations faced by rural mothers, which led them to forgo luxury services such as food, drinks, and clothing. Instead, they relied primarily on their insurance cards to cover expenses, including the purchase of drugs, even if they were not immediately available at the hospital. They would patiently wait until the required medication was in stock.

### Limitation of the study

This study might subjected to some recall bias as relied on self-reported interview and those participants stayed for long days might forget how much they paid. Thus, income may be overstated or underestimated.

## Conclusion and recommendation

The finding of this study concludes that the median hidden cost of hospital-based delivery was relatively high. Residence, family monthly income, obstetrics complication, distance from health facility and mode of delivery were significantly associated with hidden costs of hospital based delivery. Direct medical cost was the leading cost of hidden expenses followed by indirect cost.

### Recommendations

#### For policy makers

Implement income-based financial assistance programs to support expectant parents with lower monthly incomes. These programs can provide subsidies or discounts on hospital charges, medications, and other related expenses, reducing the financial burden for families with limited financial resources.

#### For zonal and woreda transportation bureau

Provide transportation support or arrange affordable transportation options for expectant mothers residing in remote or distant areas. This can help alleviate the financial burden of traveling to the hospital for childbirth and encourage timely access to healthcare services.

#### For health professionals

It is recommended that all healthcare professionals consistently adhere to antiseptic techniques, provide timely administration of pain relief and antibiotics, conduct smart risk assessments, and ensure prompt referral services for patients. These measures are crucial in preventing obstetric complications that can prolong hospital stays and result in increased healthcare expenditures.

### Electronic supplementary material

Below is the link to the electronic supplementary material.

Supplementary Material 1

## Data Availability

All necessary data were included in the paper. However, the raw dataset used in the current study are not available to the general public. Since not all participants gave their permission for us to publish the raw data, but they are still available upon reasonable request from the corresponding author (Menen Tilahun).

## Abbreviations

EMONC: Emergency Obstetric and New Born Care
ETB: Ethiopian Birr
EDHS: Ethiopia Demographic Health Surveillance
FMOH: Federal Ministry of Health
GDP: Gross Domestic Product
MHC: Maternal Health Care
MMR: Maternal Mortality Ratio
SD: Standard Deviation
SNNPR: Southern Nation Nationalities and People Region
SPSS: Statistical Package for Social Sciences
