# Supplementary material for: Hidden cost of hospital-based delivery and associated factors among postpartum women attending public hospitals in Gamo zone, southern Ethiopia
Source: BMC Health Serv Res. 2024 Apr 22;24:495. doi: 10.1186/s12913-024-10927-y (PMC11034158; doi:10.1186/s12913-024-10927-y)
Supplement: Supplementary file 1 — Supplementary Material 1 [file 12913_2024_10927_MOESM1_ESM.docx]

## Annexes II: English version questionnaire

**Part I: Socio- demographic characteristics.**

| **s.no** | **Question** | **Category/answer** | **skip** |
| --- | --- | --- | --- |
| 101 | What is your age | **………………….** |  |
| 102 | From where residence did you come? | 1.urban  2.rural |  |
| 103 | By what system you come here? | 1.refereed from health center  2. by your self |  |
| 104 | What religion do you belong to? | 1.orthodox  2.musilim  3.protestant  4.Others |  |
| 105 | Do you know write and read | 1.yes  2.no |  |
| 106 | If yes, what is the level of education  that you have attained | 1.no formal education  2.primary education  3 secondary education  4, more than secondary education……….. |  |
| 107 | Does your husband write and read? | 1.yes  2.no |  |
| 108 | If yes, what is the level of education  that you have attained | 1.no formal education  2.primary education  3 secondary education  4,more than secondary education |  |
| 109 | What is your present occupation? | 1.prievate employee  2.government employee  3.merchant  4.house wife |  |
| 110 | What is the present occupation of your  Husband? | 1.prievate employee  2.government employee  3.merchant  4.farmer |  |
| 111 | How many individuals are there in the  Household? |  |  |
| 112 | What is the average monthly income of  Your family? | family**….**birr |  |

**Part II: Delivery Related information**

| **S. No** | | **Questions** | | **Response** | **Skip to** |
| --- | --- | --- | --- | --- | --- |
| 201 | | Which number of pregnancy is it? | | 1,once  2, two  3,three  4,four and above |  |
| 202 | | By what method did you deliver? | | 1 vaginal delivery  2,C-section |  |
| 203 | | How long it take to you reach this hospital? | | By KM _____________ |  |
| 204 | | How many days did you stay this hospital? | | ________ days _______ hours |  |
| 205 | Place of delivery | | 1.General hospital  2.primary hospital | |  |
| 206 | Do you face a complication in your hospital stay | | 1. yes  2.no | |  |

**Part III: Maternity care expenditure**

**Direct medical cost**

| **S. No** | **Questions** | **Response** | **Skip to** |
| --- | --- | --- | --- |
| 301 | Do you pay for medicine purchase from private pharmaceutical? | 1, No  2, yes | If no skip to Q 18 |
| 302 | If yes, Q 301 how many cost did you incurred for medicine? | ____________ETB |  |
| 303 | Do you pay for prescribed medication from hospital? | 1, N0  2, yes |  |
| 304 | If yes, Q 303 how many cost did you incurred for medicine? | ___________ETB |  |
| 305 | Do you pay for other medical equipment such as Sutures, IV Fluid etc.? | 1,No  2,Yes |  |
| 306 | If yes, Q 305 how many cost did you incurred for this equipment? | ___________ETB |  |
| 307 | Do you pay for Operation procedure during staying here? | 1, No  2, Yes |  |
| 308 | If yes, Q 307 how many cost did you incurred for Operation procedure? | ___________ ETB |  |
| 308 | Do you pay for Bed service during staying here? | 1, No  2, yes |  |
| 309 | If yes Q 308 how many cost did you incurred for Bed service? | ___________ ETB |  |
| 310 | Do you pay for Dressing and Bandages? | 1, No  2, Yes |  |
| 311 | If yes, Q 310 how many cost did you incurred for Dressing and bandages? | ___________ ETB |  |
| 312 | Do you pay for needle and syringes during staying here? | 1, No  2, Yes |  |
| 313 | If yes, Q 312how many cost did you incurred for needle and syringes? | ____________ETB |  |
| 314 | Other (please specify )_____________ | ____________ETB |  |

**Part IV: Direct non-medical cost**

| I will ask you about the costs of your traveling to health facilities visited during delivery | | | | | | | | | | | | | |
| --- | --- | --- | --- | --- | --- | --- | --- | --- | --- | --- | --- | --- | --- |
| 401 | Did you use transport to come Hospital? | 1, No  2, Yes | | | | | | | | | | If no Skip to Q 35 | |
| 402 | If yes Q 401 what transport modality  Have you used to travel from home to Hospital (H→H)? | 1, Ambulance  2, Private car  3, Bajaj  4, Other (specify………) | | | | | | | | | |  | |
| 403 | Is there any cost incurred for transport to come hospital? | 1, No  2, Yes | | | | | | | | | |  | |
| 404 | If yes, Q 403 How many cost have you incurred to travel from Home to Facility? | No of person | | | Individual  cost | | | Total cost | | | |  | |
|  |  | ____ | | | _____ETB | | | ____ETB | | | |  |  |
| 405 | What is transport modality have you used or intend to use for return trip? | 1, Ambulance  2, Private car  3, Bajaj  4, Other (specify…………………) | | | | | | | | | |  | |
| 406 | Is there any cost will you incurred for return trip? | 1, No  2, Yes | | | | | | | | | | If no skip to Q 38 | |
| 407 | If yes, Q 406 how much cost have you Incurred or will you incur for return trip? | No of person | | Individual  Cost | | | | | Total cost | | |  | |
|  |  | ____ETB | | _____ETB | | | | | ____ ETB | | |  |  |
| 407 | Is there any cost incurred for food during staying here? | 1, No  2, Yes | | | | | | | | | | If no skip to Q 40 | |
| 408 | If yes, Q 407 How much cost for food did you incurred during staying here? | No of person | Individual  Cost | | | | No of day  of stay | | | Total cost | |  | |
|  |  |  | **…………..** | | | | **…….** | | | ____ ETB | |  |  |
| 409 | Is there any cost incurred for drinking during staying here? | 1= No  2= Yes | | | | | | | | | | If no skip to Q 42 | |
| 410 | If Yes, Q 409how much cost for drinking did you incurred during staying here? | No of person | Individual  cost | | | No of day  of stay | | | | | Total cost |  | |
|  |  | **……..** | **……….** | | | **……….** | | | | | ____ ETB |  |  |
| 411 | Did you use rent bed during staying here? | 1, No  2, Yes | | | | | | | | | | Care giver | |
| 412 | If Q 411 yes, how much cost did you pay per day? | ___________ ETB | | | | | | | | | |  | |
| 413 | Did you incur cost for communication? | 1, No  2, Yes | | | | | | | | | |  | |
| 414 | If Q 413 yes, How much cost did you incurred? | ____________________ ETB | | | | | | | | | |  | |
| **Indirect cost** | | | | | | | | | | | | | |
| 501 | Did you get monthly income? | 1.No  2.yes | | | | | | | | | | |  |
| 502 | If yes how much you get in a month. | …………………ETB | | | | | | | | | | |  |
| 503 | Have you been stayed from the beginning at hospital? | 1, No  2, Yes | | | | | | | | | | |  |
| 504 | If yes, how many days in total did you stay at the hospital? | ___________________ Days | | | | | | | | | | |  |
| 505 | Averagely how many wage loss due to staying here? | ____________________ ETB | | | | | | | | | | |  |
| 506 | Is there any cost except from the above? | 1, No  2, Yes | | | | | | | | | | |  |
| 507 | If yes, other(please specify)_____________ | _____________________ ETB | | | | | | | | | | |  |
